# HNF4alpha Dysfunction as a Molecular Rational for Cyclosporine Induced Hypertension

**DOI:** 10.1371/journal.pone.0016319

**Published:** 2011-01-27

**Authors:** Monika Niehof, Jürgen Borlak

**Affiliations:** 1 Center of Molecular Medicine and Medical Biotechnology, Fraunhofer Institute for Toxicology and Experimental Medicine, Hannover, Germany; 2 Center of Pharmacology and Toxicology, Medical School of Hannover, Hannover, Germany; University of Illinois at Chicago, United States of America

## Abstract

Induction of tolerance against grafted organs is achieved by the immunosuppressive agent cyclosporine, a prominent member of the calcineurin inhibitors. Unfortunately, its lifetime use is associated with hypertension and nephrotoxicity. Several mechanism for cyclosporine induced hypertension have been proposed, i.e. activation of the sympathetic nervous system, endothelin-mediated systemic vasoconstriction, impaired vasodilatation secondary to reduction in prostaglandin and nitric oxide, altered cytosolic calcium translocation, and activation of the renin-angiotensin system (RAS). In this regard the molecular basis for undue RAS activation and an increased signaling of the vasoactive oligopeptide angiotensin II (AngII) remain elusive. Notably, angiotensinogen (AGT) is the precursor of AngII and transcriptional regulation of AGT is controlled by the hepatic nuclear factor HNF4alpha. To better understand the molecular events associated with cyclosporine induced hypertension, we investigated the effect of cyclosporine on HNF4alpha expression and activity and searched for novel HNF4alpha target genes among members of the RAS cascade. Using bioinformatic algorithm and EMSA bandshift assays we identified angiotensin II receptor type 1 (AGTR1), angiotensin I converting enzyme (ACE), and angiotensin I converting enzyme 2 (ACE2) as genes targeted by HNF4alpha. Notably, cyclosporine represses HNF4alpha gene and protein expression and its DNA-binding activity at consensus sequences to AGT, AGTR1, ACE, and ACE2. Consequently, the gene expression of AGT, AGTR1, and ACE2 was significantly reduced as evidenced by quantitative real-time RT-PCR. While RAS is composed of a sophisticated interplay between multiple factors we propose a decrease of ACE2 to enforce AngII signaling via AGTR1 to ultimately result in vasoconstriction and hypertension. Taken collectively we demonstrate cyclosporine to repress HNF4alpha activity through calcineurin inhibitor mediated inhibition of nuclear factor of activation of T-cells (NFAT) which in turn represses HNF4alpha that leads to a disturbed balance of RAS.

## Introduction

Cyclosporine is a potent immunosuppressive agent and widely used in transplantation medicine and in the treatment of several autoimmune diseases. However, it is known for a long time that its clinical application is confounded by unwanted secondary effects, notably new-onset diabetes, renal dysfunction, renal vascular damage and arterial hypertension [1]–[4]. A systematic review of cyclosporine's effects on blood pressure was recently reported [5]. There is definitive proof for cyclosporine to increase blood pressure in a dose-related fashion and was associated with an increased risk of stroke, myocardial infarction and heart failure. Likewise, cyclosporine-induced hypertension was observed in various animal models in vivo, e.g. in mouse [6], rats [7]–[12], dogs [13], [14], sheep [15], and primates [8], [16]. Several mechanism, including activation of the sympathetic nervous system, endothelin-mediated systemic vasoconstriction, impaired vasodilatation secondary to reduction in prostaglandin and nitric oxide, altered cytosolic calcium translocation, and activation of the renin-angiotensin system (RAS) have been proposed to underlie cyclosporine-induced hypertension [17]–[19]. Notably, the RAS system is a coordinated hormonal cascade playing a key role in the regulation of blood pressure with the peptide angiotensin II (AngII) as principle effector. Cyclosporine was reported to elevate RAS components in transplant patients, e.g. plasma renin activity [20]–[22], AngII levels [20]–[22], angiotensin converting enzyme (ACE) activity [23], [24], or angiotensin receptors (AGTR1) [25]–[28], even though the effects of cyclosporine on RAS in man are to some extend contradictory, since normal or even lower plasma renin activity had been reported as well [18], [29]–[31]. However, the lack of increase in plasma renin activity in some clinical studies does not exclude activation of tissue RAS, which plays additional important functions but is not necessarily seen as a change in plasma renin activity [31]–[33]. Furthermore, cyclosporine also exerts structural nephrotoxicity which may further increase plasma renin activity [18], [25], [34]. Thus, Ras activation may be both a cause and a consequence of cyclosporine-induced renal damage [18]. Nevertheless, cyclosporine induced blood pressure changes occur prior to renal damage [18]. Diverse antihypertensive drugs are available to treat high blood pressure and clinical trials evidenced the benefit of inhibitors of RAS, i.e. ACE inhibitors and AGTR blockers for the prevention of cardiovascular diseases in the general population [35] as well as in transplant recipients [36]. While several mechanisms including RAS activation had been discussed as possible cause for cyclosporine induced hypertension, a detailed molecular rational has not been proposed as yet.

Recently, we proposed cyclosporine to repress HNF4α/HNF1α and subsequent regulation of genes coding for glucose metabolism and of pancreatic beta-cell function as a molecular rational for posttransplantation diabetes mellitus, which is an other acknowledged complication in calcineurin inhibitor immunosuppressive therapies [37]. HNF4α is a master regulatory protein in liver biology and an important transcription factor in angiotensinogen (AGT) gene regulation [38]. Specifically, AGT is synthesized in the liver and secreted into the circulation [39]. It is the substrate of renin, the rate-limiting enzyme of the RAS enzymatic cascade that generates the decapeptide angiotensin I (AngI), which then becomes further processed to the functional vasoactive octapeptide AngII by activity of the angiotensin I converting enzyme (ACE). It is known that variation in AGT transcription influences control of blood pressure [38]. Here, we studied the effect of cyclosporine on HNF4α and various RAS components to better understand cyclosporine induced hypertension and proposed HNF4α dysfunction and subsequent regulation of AGTR1, ACE, and ACE2 as a molecular rational.

## Materials and Methods

### Cell culture experiments

The human hepatoma cell line HepG2 was obtained from LGC-ATCC Standards (Wesel, Germany). In a dose-range finding study concentrations of up to 30 µg/ml were tested for cell viability with the MTS assay (CellTiter 96® Aqueous One Solution Cell Proliferation Assay, Promega, Mannheim, Germany). Thus, HepG2 cells were treated daily with 10 µg/ml cyclosporine (Sandimmun, Novartis, Nürnberg, Germany) for 72 h that represents 8 to 10 times of the Cmax seen in patients [40]. Treatment started at 40-50% confluence. Additionally, HepG2 cells were treated with a combination of cyclosporine and captopril at 10 µg/ml for 72 h, or alternatively for 4 h with captopril alone after treatment with cyclosporne for 72 h.

Additionally, experiments were carried out with primary human hepatocytes. Notably, liver specimens were obtained from patients undergoing hepatic resections at the Medical School of Hannover, Germany. Written approval for the use of human liver specimens was obtained from patients undergoing hepatic resections with approval of the ethics committee of the Medical School of Hannover. Details regarding the isolation and culture of human hepatocytes in the collagen sandwich were previously described [41].

### Isolation of nuclear extracts, western blotting analysis and electrophoretic mobility shift assays

The experimental procedures for nuclear extracts isolation, western blotting analysis and electrophoretic mobility shift assays (EMSAs) are given in Niehof and Borlak, 2009 [42]. The antibodies directed against HNF4α (sc-6556x), Actin (sc-1616), Angiotensinogen (sc-7419), and AGTR1 (sc-1173) were purchased from Santa Cruz Biotechnology (Heidelberg, Germany). Nuclear extracts were prepared in triplicate and used as described in the figure legend. The antigen-antibody complexes were visualized using the enhanced chemiluminescence (ECL) detection system (PerkinElmer Life Sciences, Rodgau-Juegesheim, Germany). Light signal detection was done with the CCD camera Imager system Kodak IS 440 CF (Kodak, Biostep GmbH, Jahnsdorf, Germany) and quantification was performed using the Kodak 1D Image analysis software (version 3.5.). The oligonucleotides (for sequence information see Table 1) were purchased from MWG Biotech (Ebersberg/Muenchen, Germany), annealed and ^32^P-labeled. Supershift experiments were done with HNF4α specific antibody (sc-6556x).

**Table 1:** Shift-probe sequences.

Gene	Oligo name	Accession number	Sequence
HNF1α	HNF1	NM_000545	AGGGCTGAAGTCCAAAGTTCAGTCCCTTC
AGT	AGT	NM_000029	TGCAGAGGGCAGAGGGCAGGGGA
AGTR1	AGTR1	NM_000685	TAAGGAGGGCAAAGTTCAAGTGA
ACE	ACE1	NM_000789	TGGGCCTACCAAAGGTCAGGAGA
ACE2	ACE2(a)	NM_021804	AGTCTATGTACTTTGCTCTAGCA
ACE2	ACE2(b)	NM_021804	CCGGAGTGATCTTTGACTCTTTC

### Quantitative real-time RT-PCR

Quantitative real-time RT-PCR measurement was done with the Lightcycler (Roche Diagnostics, Mannheim, Germany) with the following conditions: denaturation at 95°C, annealing at different temperatures for 8 sec, elongation at 72°C for different times and detection of SYBR-Green I-fluorescence at different temperatures. Detailed primer specific conditions and oligonucleotide sequence information are given in Table 2. Relative quantification was performed using the "Fit Points Method" of the LightCycler3 Data Analysis Software version 3.5.28 (Roche Diagnostics, Mannheim, Germany) by comparing the sample values to a standard curve within the linear range of amplification. The standardized sample values for each gene of interest were divided by the standardized values of the housekeeping gene (mitATPase), which was found to be stably expressed.

**Table 2:** Real-Time PCR primer sequences and amplification settings.

Gene	Accession number	Primer sequence	Fragment length	Annealing	Elongation	Fluorescence
HNF4α	NM_000457	fwd: CTGCTCGGAGCCACAAAGAGATCCATGrev: ATCATCTGCCACGTGATGCTCTGCA	371bp	60°C	15sec	88°C
AGT	NM_000029	fwd: GGATGAGAGAGAGCCCACAGrev: CTCACTCCATGCAGCACACT	351bp	68°C	15sec	90°C
ACE	NM_152830	fwd: ATGTAGATGCAGGGGACTCGrev: CCAGGGAGGTGAAGAAATCA	342bp	60°C	14sec	88°C
ACE2	NM_021804	fwd: GATCCCATGGCTACAGAGGArev: GCCAGGAAGAGCTTGACATC	228bp	60°C	20sec	82°C
AGTR1	NM_000685	Exon2/Exon5fwd: CTGATGCCATCCCAGAAAGTrev: CTTCCAGCTTTGGGACAATC	144bp	60°C	20sec	78°C
		Exon5/Exon5fwd: CAAGACAAAGCAAAGCCACArev: CAGGACAAAAGCAGGCTAGG	136bp	60°C	20sec	78°C
mitATPase	NC_012920	fwd: CTAAAGGACGAACCTGArev: TGGCCTGCAGTAATGTT	315bp	55°C	13sec	83°C

### Statistical analysis

Experiments are performed at least in triplicate. All values are expressed as mean ± standard deviation. To determine significance between two groups, comparison was made using the non-parametric two-tailed Mann-Whitney-U-Test. Therefore, Statistica software, version 8 (StatSoft) was used. The results are considered significant when the p value was less than 0.05.

## Results

### Gene expression of members of the renin-angiotensin system after cyclosporine treatment

**Figure 1:**
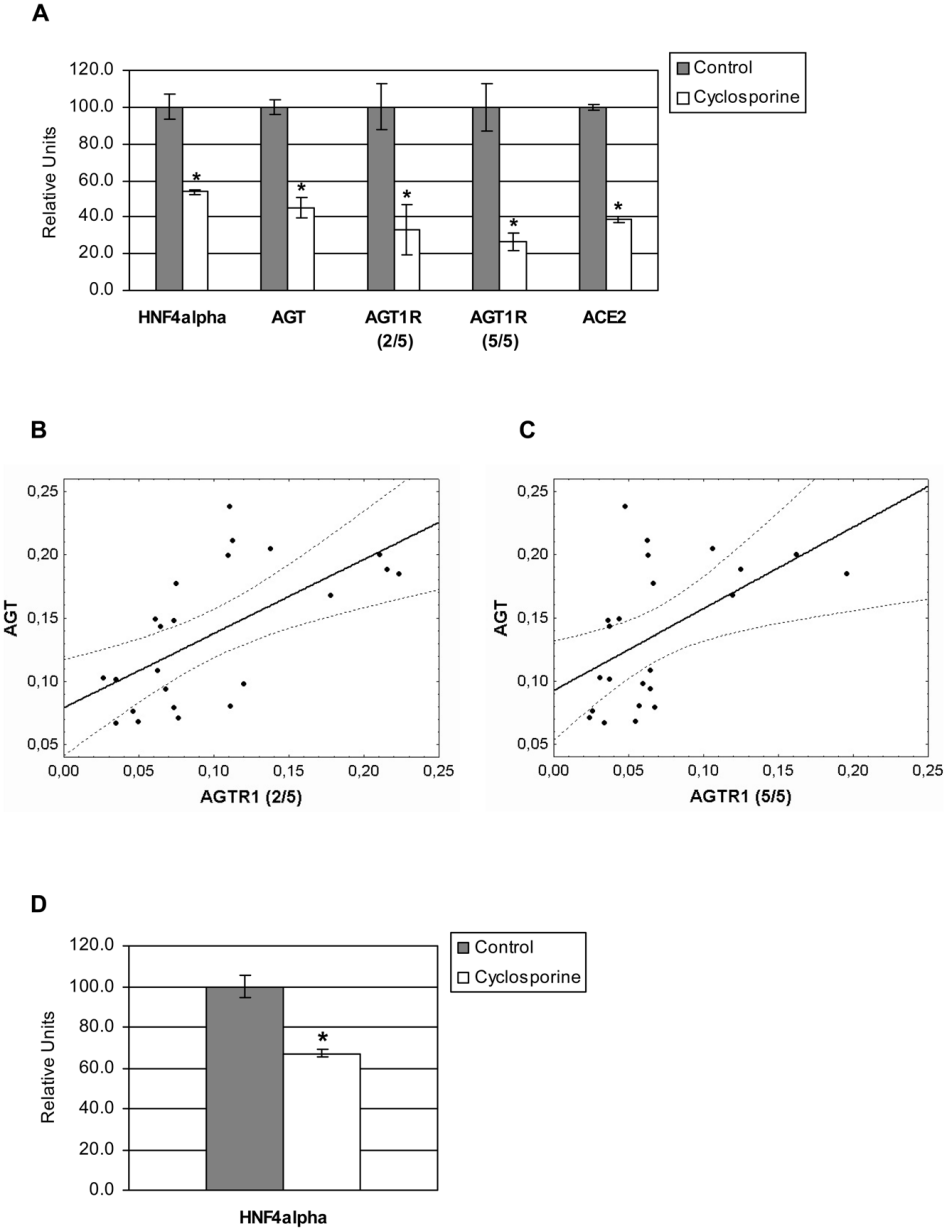
Regulation of gene expression in HepG2 cells after cyclosporine treatment. A. Gene expression was measured by quantitative real-time RT-PCR in HepG2 cells 72 h after treatment with 10 µg/ml cyclosporine (n = 3, respectively) and was determined relative to expression of mitATPase6, which served as a housekeeping gene. Gene expression in untreated HepG2 cells was set to 100 and values for cyclosporine treatment represent transcript abundance relative to control. Non-parametric Mann-Whitney-U-Test was used to compare cyclosporine treated and control groups. Results are considered significant at p<0.05 (marked with an asterik). AGT (angiotensinogen), AGTR1 (angiotensin II receptor, type 1), 2/5 (Primer Exon 2/Exon 5), 5/5 (Primer Exon 5/Exon 5), ACE2 (Angiotensin I converting enzyme 2). B and C. Gene expression of AGT and AGTR1 (2/5) (B) or AGTR1 (5/5) (C) in HepG2 cells. Gene expression was determined by quantitative real-time RT-PCR in untreated HepG2 cells (n = 11) and in cyclosporine treated HepG2 cells (n = 12). Linear regression of correlation with 0.95 confidence interval are shown. AGTR1 (2/5): r^2^ = 0.3910, p = 0.0014; AGTR1 (5/5): r^2^ = 0.2688, p = 0.0113. AGT (angiotensinogen), AGTR1 (angiotensin II receptor, type 1), 2/5 (Primer Exon 2/Exon 5), 5/5 (Primer Exon 5/Exon 5). D. Gene expression of HNF4α was measured by quantitative real-time RT-PCR in total RNA extracts obtained from cultures of primary human hepatocytes treated with 30 µg/ml cyclosporine for 72h in n = 3 replicates measurements. Expression of mitATPase6 served as a housekeeping gene. Gene expression in untreated human hepatocytes was set to 100 and values for cyclosporine treatment represent transcript abundance relative to control. Non-parametric Mann-Whitney-U-Test was used to compare cyclosporine treated and control groups. Results are considered significant at p<0.05 (marked with an asterik).

Angiotensinogen, a member of the renin-angiotensin system (RAS), is mainly synthesized in the liver [38]. We therefore used the HepG2 human hepatoma cell line as model system. Initially, we studied expression of HNF4α and of members of the RAS cascade in cyclosporine treated HepG2 cells. By quantitative real-time RT-PCR we found HNF4α, angiotensinogen (AGT), angiotensin receptor 1 (AGTR1), and angiotensin converting enzyme 2 (ACE2) to be significantly repressed after treatment with 10 µg/ml cyclosporine for 72 h (Fig. 1A). Expression of renin and angiotensin converting enzyme (ACE) was not detected in HepG2 cells.

Furthermore, at least five splice variants have been identified for the AGTR1 protein, but only exon 5 encodes the open reading frame [43], [44]. Therefore, we analyzed AGTR1 transcript splice variants and evidenced expression of AGTR1 for exon 2 [AGTR1 (2/5), Fig. 1A] and exon 5 [AGTR1 (5/5), Fig. 1A] in untreated HepG2 cells; while expression of exon 3 and exon 4 was not detected. After treatment of HepG2 cells with cyclosporine expression of AGTR1 was significantly repressed, but isoform expression did not change (Fig. 1A). The gene expression of AGT and AGTR1 was significantly correlated when either untreated or treated HepG2 cells are studied (Fig. 1B, C).

The consequences of drug treatment with the ACE inhibitor captopril was investigated. For this purpose, HepG2 cells were treated for 72 h with a combination of cyclosporine and captopril (10 µg/ml) or alternativly for 4 h with captopril alone after treatment with cyclosporine for 72 h. Repressed gene expression of HNF4α, AGT, and AGTR1 by cyclosporine remained unchanged after captopril treatment under both conditions (Table 3).

**Table 3:** Gene expression in HepG2 cells after treatment with captopril.

Gene	Treatment	Transcript abundance(relative units, mean ± SD)	% of control
HNF4α	Control	1.846±0.127	100
	Cyclosporine	0.993±0.024	53.8
	Cyclosporine + Captopril, 72h	1.204±0.138	65.2
	Cyclosporine + Captopril, 4h	1.106±0.053	59.9
AGT	Control	0.186±0.026	100
	Cyclosporine	0.071±0.005	38.2
	Cyclosporine + Captopril, 72h	0.078±0.007	41.9
	Cyclosporine + Captopril, 4h	0.073±0.005	39.2
AGTR1	Control	0.335±0.185	100
	Cyclosporine	0.075±0.010	22.4
	Cyclosporine + Captopril, 72h	0.087±0.022	26.0
	Cyclosporine + Captopril, 4h	0.095±0.006	28.4

Gene expression was measured by quantitative real-time RT-PCR in HepG2 cells after treatment with 10 µg/ml cyclosporine (n = 3, respectively) for 72 h. Furthermore, cells were treated in parallel with 10 µg/ml ACE inhibitor captopril (cyclosporine + captopril, 72h), or with cyclosporine for 72h and subsequently with 10 µg/ml captopril for 4 h (cyclosporine + captopril, 4h). Transcript abundance of HNF4α, AGT, and AGTR1 are determined relative to expression of mitATPase6, which served as a housekeeping gene. Data are given as relative units (mean ± SD). Furthermore, transcript abundance of HNF4α, AGT, and AGTR1 in untreated HepG2 cells was set to 100 and values for cyclosporine or cyclosporine/captopril treatment represent transcript abundance relative to the control.

To confirm results obtained with HepG2 cell cultures of primary human hepatocytes were treated with cyclosporine as well. As observed with the HepG2 cell line HNF4α gene expression was significantly repressed in cultures of primary human hepatocytes after treatment with cyclosporine (Fig. 1D).

### Cyclosporine inhibits binding of HNF4α to members of the renin-angiotensin system

**Figure 2:**
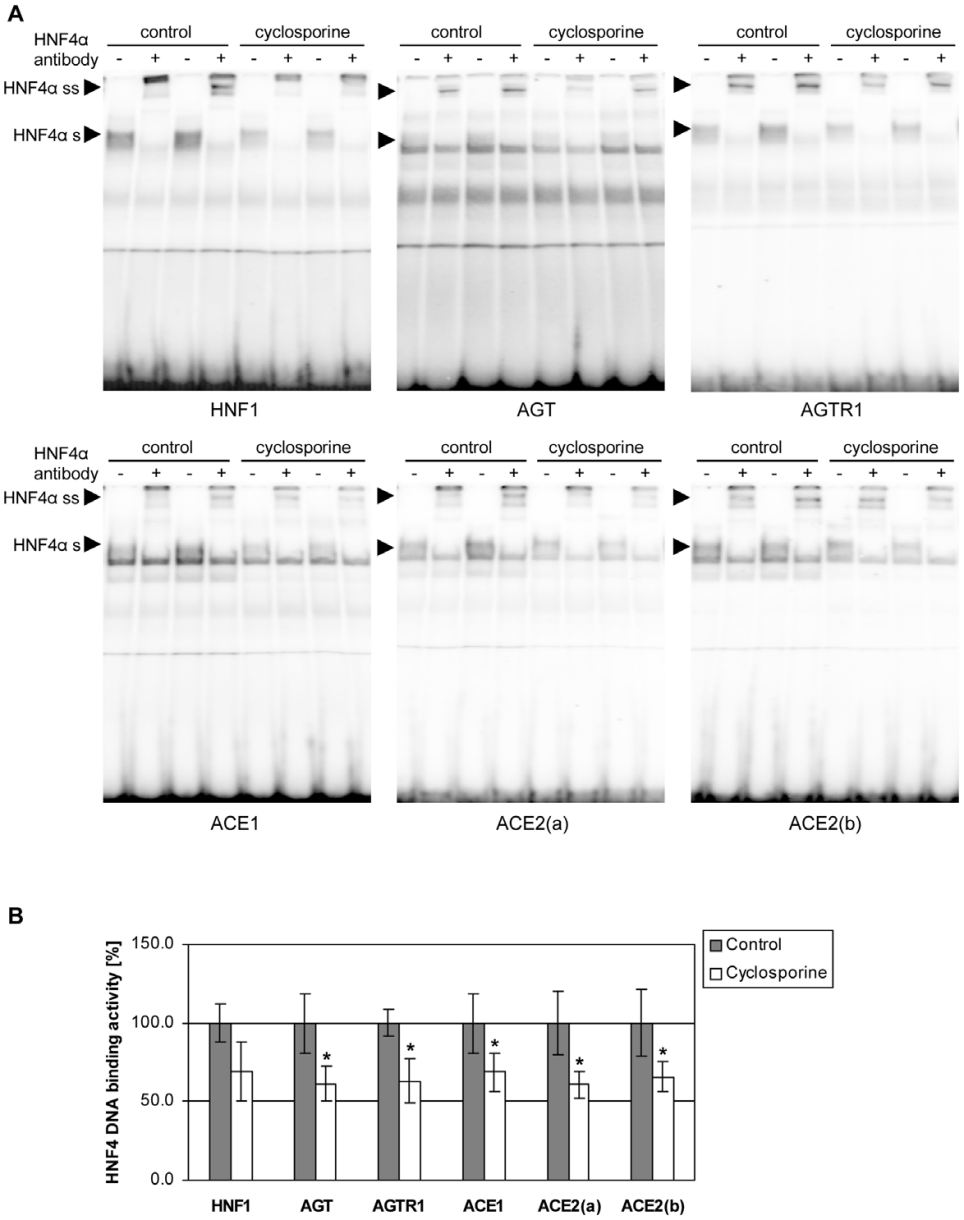
Cyclosporine inhibits binding of HNF4α to target genes. A. Electrophoretic mobility shift assays with 5 µg HepG2 cell nuclear extract [control or cyclosporine treatment, 10 µg/ml for 72 h, n = 3, respectively] and ^32^P labeled oligonucleotides to probe for DNA binding to HNF4α binding-sites within HNF1α (HNF1), angiotensinogen (AGT), angiotensin II receptor, type 1 (AGTR1), angiotensin I converting enzyme (ACE1), and angiotensin I converting enzyme 2 [ACE2(a), ACE2(b)] genes. In EMSA supershift assays an antibody directed against HNF4α (+) was added. Shifted (HNF4α s) and supershifted bands (HNF4α ss) were marked. B. Dried EMSA gels were analyzed with a Molecular Imager (BioRad, Muenchen, Germany) using the Quantity One software (BioRad, Muenchen, Germany). HNF4α binding of control extracts to the respective binding sites was set to 100% and inhibition of binding to the respective binding sites after treatment with cyclosporine (10 µg/ml for 72 h) was quantified. Non-parametric Mann-Whitney-U-Test was used to compare cyclosporine treated and control groups. Results are considered significant at p<0.05 (marked with an asterisk).

It is known that HNF4α binds to promoter elements of AGT and strongly activates its transcription [38]. We therefore investigated HNF4α DNA binding activity after cyclosporin treatment. Importantly, EMSA provided evidence for a cyclosporine dependent decrease in HNF4α DNA-binding to AGT as well as to the A-site of the HNF1α promoter (HNF1), i.e. an additional established recognition site for HNF4α (Fig. 2A, B). We then searched for HNF4α binding sites in proximal promoter regions of further members of the renin-angiotensin system. Essentially, the transcription start site (TSS, +1) of the NCBI mRNA reference sequence (RefSeq) was aligned using the UCSC Genome Browser (http://genome.ucsc.edu/) for promoter annotation of the respective genes. Proximal promoters (up to –10000 bp) of human ACE and ACE2, and intronic regions of human AGTR1 were checked for putative HNF4α binding-sites with the tool MATCH [45], by employing the Transfac weight matrix V$HNF4_Q6_01 (generated by Biobase, www.biobase.de). We detected binding sites within the AGTR1, ACE and ACE2 gene. Binding site positions and scores for matrix similarity and core similarity are indicated in Table 4. The ability of HNF4α to bind to recognition sites was studied with 32P-labeled probes encompassing predicted HNF4α-sites located in the AGTR1, ACE, and ACE2 [ACE2(a) and ACE2(b)] gene. Predicted binding sites were confirmed by EMSA bandshift assays using an antibody specific for HNF4α. We observed strong binding of nuclear extracts of untreated cell cultures to all predicted sites (Fig. 2A). Addition of a specific HNF4α antibody shifted the bands, therefore providing clear evidence for the specificity and selectivity of the assay. Strikingly, cyclosporine significantly reduced binding of HNF4α to all EMSA probes employed to approximately 60% when compared to untreated cell cultures (Fig. 2B).

**Table 4:** Predicted binding sites for HNF4α in different genes of the renin-angiotensin system.

Gene name	Oligo name	Accession number	bp relative to TSS^a^	Sequence	Matrix	Score core/matrix
AGTR1	AGTR1	NM_000685	+39426 to +39439	GAGGGCAAAGTTCA	V$HNF4_Q6_01	1.000/1.000
ACE	ACE1	NM_000789	−4690 to −4668	CTACCAAAGGTCAG	V$HNF4_Q6_01	1.000/0.909
ACE2	ACE2(a)	NM_021804	−4380 to −4358	ATGTACTTTGCTCT	V$HNF4_Q6_01	1.000/0.969
ACE2	ACE2(b)	NM_021804	−6283 to −6261	GTGATCTTTGACTC	V$HNF4_Q6_01	1.000/0.896

a TSS  =  transcription start site, NCBI GenBank Version Build 36.1 (hg18).

### Cyclosporine inhibits protein expression of HNF4α

**Figure 3:**
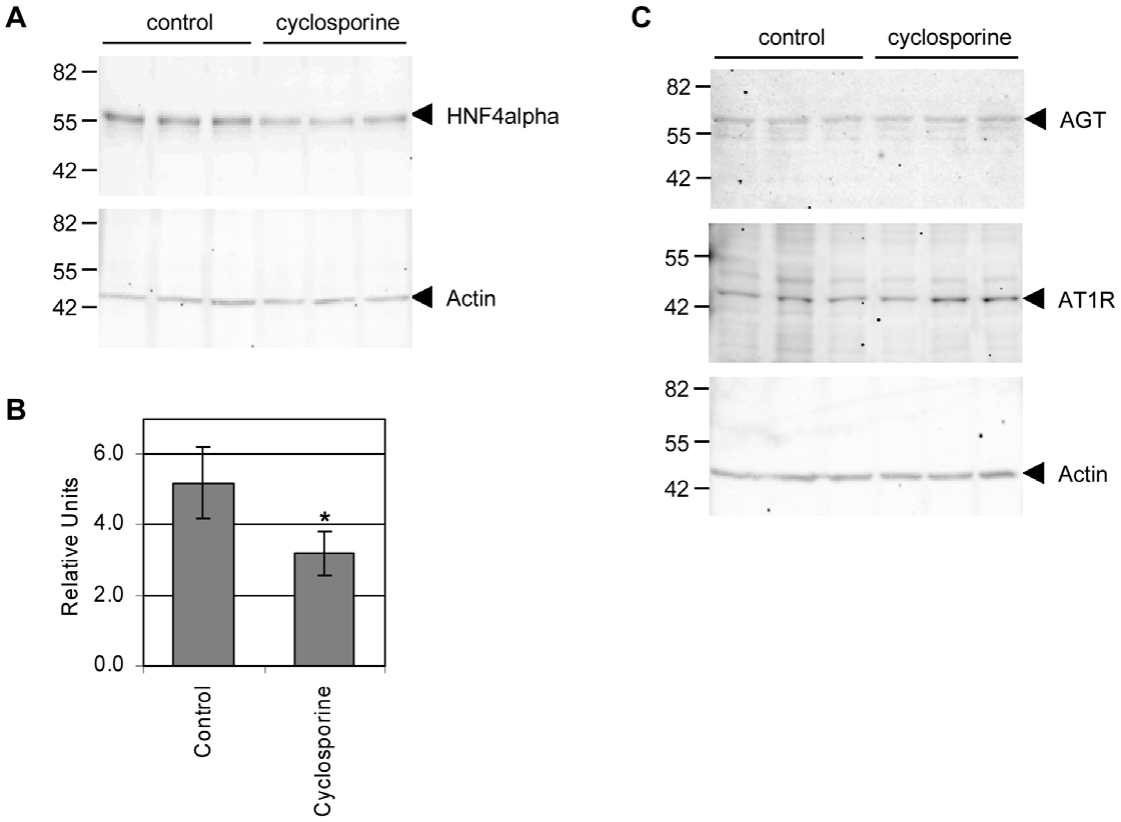
Cyclosporine inhibits protein expression of HNF4α. A. HNF4α western blotting of 20 µg HepG2 cell nuclear extracts (upper panel) and actin western blotting (lower panel) of 10 µg HepG2 cell nuclear extracts [control or cyclosporine treatment, 10 µg/ml for 72 h, n = 3, respectively]. B. The graph panel represent the quantification of protein amounts for HNF4α relative to the actin expression. Non-parametric Mann-Whitney-U-Test was used to compare cyclosporine treated and control groups. Results are considered significant at p<0.05 (marked with an asterisk). C. AGT, AGTR1, and actin western blotting of 30 µg, 20 µg and 15 µg total cellular extract, respectively, isolated from control or cyclosporine treated HepG2 cells (10 µg/ml for 72 h, n = 3, respectively).

HNF4α protein expression was significantly repressed after treatment of HepG2 cells with 10 µg/ml cyclosporine for 72 h (Fig. 3A). For comparison Western blotting of actin was used as housekeeping protein and to control loading onto the gel (Fig. 3A, B). Protein expression of AGT and AGTR1 remained unchanged after treatment with cyclosporine (Fig. 3C).

## Discussion

Cyclosporine had been introduced for more then 20 years and represents a major breakthrough in transplantation medicine. Unfortunately, treatment of patients with calcineurin inhibitors results in unwanted side effects, notably hypertension and nephrotoxicity [1]–[3], [5]. Although these adverse effects are already known for a long time and several mechanism had been explored, the molecular basis for cyclosporine induced hypertension remains elusive.

Specifically, cyclosporine binds to the cyclophillin receptor. This drug-receptor complex inhibits activation of the calcineurin phosphatase, a secondary messenger in the dephosphorylation and activation of nuclear factor of activation of T-cells (NFAT). NFAT is a DNA-binding protein that promotes transcription of interleukin-2 (IL-2) but inhibition of IL-2 production stops proliferation and activation of T-cells to mediate the immunosuppressive effect of cyclosporine [46]. Indeed, activation of the RAS system is one of the important mechanism involved in cyclosporine induced hypertension and represents a coordinated hormonal cascade of major importance in the control of cardiovascular and renal tonus [18]. Cyclosporine causes RAS activation by an increase in plasma renin and ACE activity with subsequent generation of elevated AngII levels [18], [20]–[24], [34]. Furthermore, long-term treatment with cyclosporine is associate with up-regulated AGTR1 receptors in vascular and renal tissues [25]–[28]. The exact molecular causes leading to RAS activation and AngII increase are unknown; its activation is probably a multifactorial process [18]. In addition to calcineurin inhibition, cyclosporine stimulates PDGF [47] and TGFβ [48], [49] signaling that seems to be involved in mediating renin secretory effects of cyclosporine and it is well established that binding of AngII to AGTR1 leads to vasoconstriction and hypertension. Thus, an increase in blood pressure may contribute to renal damage and a subsequent increase in plasma renin activity [18]. This process may cause a vicious circle in RAS activation, which further perpetuates cyclosporine toxicity. However, cyclosporine also induces structural nephrotoxicity, which is independent of blood pressure changes [18], [25], [34]. Taken collectively, cyclosporine stimulates both, the systemic (circulating) RAS and the local (tissue) RAS components, which were identified in many organs [20]. In regards to the tissue RAS, AngII exerts further direct effects on cardiac myocytes contributing to hypertension and cardiac failure [50]. In this complex interplay HNF4α is one of the direct NFAT target genes [37], [51]. Recently, we demonstrated recently its gene and protein expression to be significantly repressed in response to cyclosporine treatment [37]. AGT is known to be regulated by HNF4α [38] and we identified AGTR1 and ACE as further HNF4α target genes among RAS members. We applied position weight matrices to predict HNF4α consensus sites and confirmed the predictions experimentally by EMSA assays to determine its DNA binding activity. Thus, we demonstrate cyclosporine treatment of human hepatoma cells to significantly reduce DNA-binding of HNF4α to AGT and AGTR1 at gene specific promoters to cause impaired gene expression. As alternative splicing might be an important mechanism in the regulation of AGTR1 expression [44] we analyzed AGTR1 isoforms before and after cyclosporine treatment. Here, expression of splice variants was unchanged in HepG2 cells. Furthermore, we analyzed the consequences of inhibition of ACE in cyclosporine treated HepG2 cells. Captopril did not influence gene expression of various RAS members in cyclosporine treated HepG2 cells. Hence, captopril selectively inhibits ACE enzymatic activity, but did not affect gene expression via feed back loops. However, RAS does not represent a linear cascade, but is composed of a sophisticated interplay between multiple mediators, receptors, and enzymes. The principal RAS effector molecule AngII modulates positive and negative feed back loops, e.g. induction of AGT [52] and a decrease in AGTR1 [44]. Furthermore, AngII acts at an additional membrane receptor, AGTR2, which antagonizes AGTR1 induced signaling thereby mediating vasodilatation, antiproliferative and apoptotic effects [53]. Importantly, RAS can be envisioned as a dual function system, in which the vasoconstrictor/vasodilator actions are primarily driven by the ACE/ACE2 balance. Indeed, ACE2, which was termed the alternative axis of RAS, efficiently hydrolyses the octapeptide AngII to Ang(1-7), a peptide that exerts actions opposite to those of AngII [54]. Ang(1-7) has protective function; it binds to the G protein coupled receptor Mas, acts as a vasodilator and antagonizes AngII-induced vasoconstriction [54]. The impact of cyclosporine on ACE2 activity or expression was not analyzed so far. Bioinformatic prediction of HNF4α binding sites and subsequent EMSA assays identified ACE2 as a further HNF4α target gene, which was significantly repressed after cyclosporine treatment of HepG2 cells. As reported elsewhere a decrease in ACE2 leads unambiguously to enhanced AngII action on AGTR1 and caused vasoconstriction and hypertension [55]. Interestingly, ACE2 is markedly reduced in rat models of hypertension [56]. It has been proposed that some of the beneficial effects of currently used protective drugs as ACE inhibitors and AGTR1 blockers are beyond their direct effects on suppression of the ACE-AngII-AGTR1 axis as they also increased Ang(1-7) production through feedback mechanisms [54]. Consequently, ACE2 may represent a novel therapeutic target within the RAS system by enhancing AngII degradation. Indeed, a potential therapeutic benefit of direct increasing ACE2 activity in the treatment of hypertension was recently suggested [55].

In conclusion, we propose cyclosporine to cause dysfunction of HNF4α which in turn disturbs the balance of the renin-angiotensin-system. Most recently, Bai et al reviewed the genetic basis for adverse drug effects of calcineurin inhibitors and proposed that the development of type 2 diabetes, hypertension, and renal failure may be associated with specific DNA genotypes [57]. They conclude that disease-associated genes may provide genomic biomarkers for exploring adverse drug effects. As there are large individual differences in HNF4α expression amongst patients as recently reported by us [37], patients with low HNF4α activity might be at higher risk for developing cyclosporine induced hypertension.
